# The efficient use of smartphone apps to improve the level of asthma knowledge

**DOI:** 10.25122/jml-2021-0367

**Published:** 2022-05

**Authors:** Muhammad Thesa Ghozali, Satibi Satibi, Zullies Ikawati, Lutfan Lazuardi

**Affiliations:** 1. School of Pharmacy, Universitas Muhammadiyah Yogyakarta, Yogyakarta, Indonesia; 2. Department of Pharmaceutics, Faculty of Pharmacy, Universitas Gadjah Mada, Yogyakarta, Indonesia; 3. Department of Pharmacology and Clinical Pharmacy, Faculty of Pharmacy, Universitas Gadjah Mada, Yogyakarta, Indonesia; 4. Department of Health Policy and Management, Faculty of Medicine, Public Health, and Nursing, Universitas Gadjah Mada, Yogyakarta, Indonesia

**Keywords:** AGKQA, asthma, asthma app, asthma knowledge, asmadroid

## Abstract

Patient education is one of the important aspects of improving knowledge and quality of asthma control. In this digital era, it can be made with the support of an app – or known as mHealth. Unfortunately, implementing applications for patient education is relatively new among asthmatic patients in Indonesia. This study aimed to determine the efficacy of the educational content of the AsmaDroid® app on the levels of asthma knowledge among asthmatic patients. This study was a randomized controlled trial carried out from December 2019 to March 2020 in the Special Region of Yogyakarta, Indonesia. A quota sampling was employed, resulting in 140 study participants being categorized into control and treatment groups. Before and after the 4-week treatment period, all participants were asked to complete a pre-test and post-test of the Asthma General Knowledge Questionnaire for Adults (AGKQA) questionnaire. All the scores were then compared to determine the efficacy of educational content on the levels of asthma knowledge. The results of descriptive statistics reported that the pretest scores of AGKQA from the control group (minimum, maximum, and mean) were 9, 25, and 19.04±2.56, respectively, and post-test scores were 10, 27, and 18.79±3.59 (p=0.47). Meanwhile, in the treatment group, these were 13, 25, and 19.11±2.87, while post-test scores were 16, 31, 23.6±3.95 (p=0.01). Additionally, there was a difference between the post-test scores of the control and treatment groups, namely 4.81 (p=0.01). The educational content of the app significantly improved the levels of asthma knowledge.

## INTRODUCTION

Asthma is a chronic medical condition generally characterized by wheezing, episodic shortness of breath, and chest tightness, particularly at night or early morning [1, 2]. When this non-communicable disease is triggered, the airway lining becomes narrow, red, and swollen, thus leading to difficulty in breathing [3, 4], with a rapid and vigorous response of the muscles, also known as hyperresponsiveness [5]. The main causes of this disease are still inconclusive; however, a recent study confirmed that genetics plays a role in its development [6]. Early intervention and effective long-term management help improve the process of airway inflammation as well as prevent possible irreversible damage. Therefore, a proper and effective asthma treatment needs to have a long-term focus on managing a long-term or chronic inflammation rather than a quick relief of the bronchial spasm [7]. Effective management is commonly associated with taking regular medications prescribed by the doctors or specialists, self-monitoring symptoms, adhering to a personalized action plan, recognizing and reducing any exposure to the potential allergens, respecting medical appointments, and communicating with professionals regarding symptoms and treatments.

The asthma management guidelines comprise four components: education regarding the disease, symptom and lung function monitoring, control of triggers or allergens, and medical treatments. According to previous studies, education consists of information regarding the disease, self-monitoring, review of regular medication, and a written action plan. The main focus of the guidelines is to control the symptoms by involving the patients in their treatment plans and execution [8, 9]. Education can improve the control of the disease with a decrease in emergency department visits and hospital admissions, thereby reducing the morbidity rate and lowering the direct and indirect costs of care [10, 11].

The implementation of apps is now rapidly becoming an integral part of this modern life, with mobile health (mHealth) used in portable devices to provide adequate knowledge of asthma self-management. In principle, the apps can help enable effective asthma self-management interventions, thereby improving the quality of life of asthmatic patients while reducing the costs for health care systems. It could potentially support self-management by overcoming the various barriers associated with the systems, enabling real-time symptom monitoring, facilitating information sharing between patients, caregivers, and health professionals, and providing helpful educational content [12]. Many asthma-related apps performed consistently well across all applied review frameworks, thereby indicating that the apps also improve asthma self-management. However, no study was conducted to examine the effectiveness of a smartphone app in improving the level of knowledge regarding asthma self-management among asthmatic patients in Indonesia. For this reason, this study primarily aimed to determine the efficacy of the educational content of the AsmaDroid^®^ app on the levels of asthma knowledge among Indonesian asthmatic patients.

## MATERIAL AND METHODS

### Study design and participants

**Figure 1:**
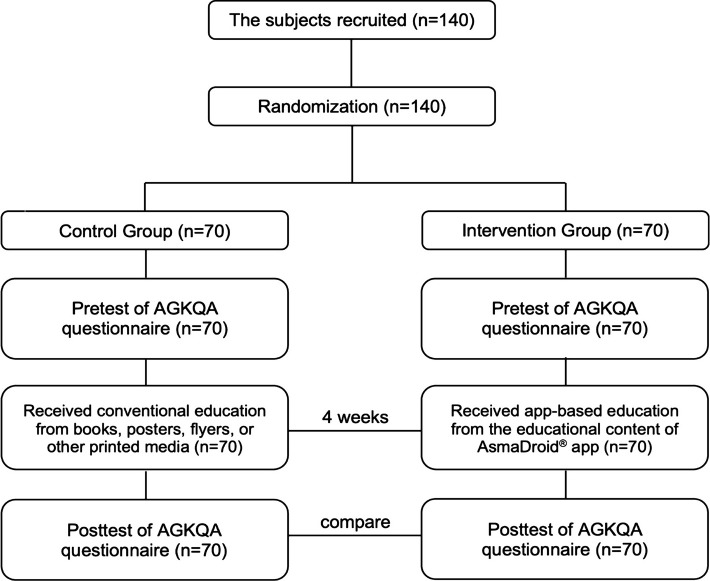
Response rate diagram of study participants.

This was a continuation of the previous study, which focused on designing and developing the AsmaDroid^®^ app [13]. This study hypothesized that the educational content of the app could significantly improve the levels of asthma knowledge among asthmatic patients. Consequently, we conducted a randomized controlled trial from December 2019 to March 2020 located in the Special Region of Yogyakarta, Indonesia. We used quota sampling and the number of participants was then calculated according to the formula by Lwanga and Lemeshow [14]. The inclusion criteria were: asthmatic patients aged 19–34 years old, having an Android smartphone, being able to download and install the AsmaDroid^®^ app, and being willing to be a participant. The exclusion criteria were respondents who failed to answer all the questions on the questionnaire and those who resigned from the study (Figure 1).

### Instruments

The instruments of this study included the AsmaDroid^®^ app and an Asthma General Knowledge Questionnaire for Adults with Asthma (AGKQA). AsmaDroid^®^ was a Google Android OS app containing educational content on asthma self-management, sourced from a combination of the Regulation of the Minister of Health of the Republic of Indonesia Number 1023 of 2008 on asthma control guidelines and official guidelines by The National Asthma Education and Prevention Program (NAEPP) Expert Panel Report 3. The educational contents included: (a) recognizing asthma, (b) classification of asthma, (c) recognizing and recommended ways to avoid the potential allergens, (c) planning long-term asthma management, (d) managing asthma attacks appropriately, (e) how to perform asthma self-monitoring, and (f) maintaining health and wellbeing. The AGKQA is a questionnaire consisting of 31 questions on general asthma knowledge with a true-and-false response format. The questionnaire was developed as one of the measures for a randomized effectiveness trial of education program for adults with asthma [15]. The list of questions in the AGKQA questionnaire was sourced from a previous study [16].

### Intervention

The intervention consisted of digital education using the AsmaDroid^®^ app, which contains patient education on asthma self-management in text and audio-video format. Technically, all the participants were categorized into two groups, *i.e*., control and treatment, with 70 participants in each group. They were asked to complete the pre-test and post-test of the AGKQA questionnaire before and after the 4-week treatment period. In the treatment group, they were briefed on using the AsmaDroid^®^ app and then asked to use it for 4 weeks. During the treatment period, they learned asthma self-management through the educational content of the app. They also received notifications to use the app. Meanwhile, the control group did not receive the app and notifications but was welcomed to independently educate themselves through conventional media, including books, posters, videos, and other media except the app. In terms of educational content, there was no difference between both groups.

### Data collection and analysis

Data were collected using the AGKQA questionnaire from December 2019 to March 2020, involving 140 participants. All participants were asked to complete the AGKQA pretest using their smartphone and then fill in the following information: (1) demographic characteristics, such as sex, age, and the levels of education, (2) experiences in using any mHealth app, and (3) scores of Asthma Control Test. After a 4-week treatment period, all participants were asked to complete the post-test questionnaire. The obtained data were further compared to determine the levels of asthma knowledge among participants from both groups. The analysis was performed using the statistical software IBM SPSS Version 22.0 for validity and reliability of the AGKQA questionnaire, descriptive analysis, and hypothesis test.

A normality test was performed before a hypothesis test to determine if the data were normally distributed. Data were normally distributed if the p-value was greater than 0.05, with more than a 95% confidence level. A Kolmogorov-Smirnov test was performed to determine the normality of the data since this study involved 140 participants. Table 1 shows the results of the Kolmogorov-Smirnov test.

**Table 1:** The results of the Kolmogorov-Smirnov test for the AGKQA questionnaire data.

Groups	Test	P-Value	Information
**Control**	Pre-test	0.120	Normally distributed
	Post-test	0.274	Normally distributed
**Treatment**	Pre-test	0.647	Normally distributed
	Post-test	0.466	Normally distribute

## RESULTS

### Study Participants

This study involved 140 patients from various groups of sex, age, education, mHealth user experience, levels of asthma knowledge, and asthma control. In this study there were more females (53.57%; n=75), aged 18–22 years old (67.85%; n=95), undergraduate (78.57%; n=110), having mHealth experiences (100%; n=140), and with high levels of asthma control (66.42%; n=93) (Table 2).

**Table 2:** Demographic characteristics of study participants.

Demographics	n (%)		P-value
	Control=70	Treatment=70	
**Sex**			
**Male**	28 (40)	37 (52.85)	0.124
**Female**	42 (60)	33 (47.15)	
**Age (years old)**			
**18–22**	47 (67.15)	48 (68.57)	0.193
**23–27**	23 (32.85)	22 (31.43)	
**Levels of Education**			
**Undergraduate**	56 (80)	54 (77.15)	0.620
**Graduate**	14 (20)	16 (22.85)	
**mHealth Experiences**			
**Yes**	70 (100)	70 (100)	-
**No**	0 (0)	0 (0)	
**Levels of Asthma Knowledge**			
**Low (<60%)**	31 (33.45)	28 (40)	0.871
**High (≥60%)**	39 (66.55)	42 (60)	
**Levels of Asthma Control**			
**Uncontrolled (<20)**	6 (8.57)	12 (17.14)	0.162
**Partially Controlled (20–24)**	45 (64.28)	48 (68.57)	
**Controlled (25)**	19 (27.15)	10 (14.29)	

### Validity and Reliability Test

A validity test is a testing model to determine how valid the instrument used to measure an object is. A reliability test mainly aims to ascertain whether the questionnaire used to collect data is reliable. In this study, the questionnaire included the Indonesian version of the Asthma General Knowledge Questionnaire for Adults (AGKQA).

A content validity test for the Indonesian version of AGKQA was conducted by a pulmonologist, a general practitioner, a pharmacist, and a nurse, who were lecturers at the Faculty of Medicine and Health Sciences, Universitas Muhammadiyah Yogyakarta. The findings of the test showed that 31 question items were good. In terms of construct validity, almost all question items (n=31) showed moderate correlation with the r-value of each item, ranging from 0.50 to 0.69. There were 6 question items with a high correlation. Table 3 comprises the results of Pearson's correlation of the Indonesian version of the AGKQA.

**Table 3:** Results of validity test of the Indonesian version of the AGKQA questionnaire.

Classification	Question (r)	Result
**Low r = 0.26–0.49** **Moderate r = 0.50–0.69** **High or strong = 0.70–0.89**	AGKQA1 (r=0.599)	Moderate Correlation
	AGKQA2 (r=0.504)	Moderate Correlation
	AGKQA3 (r=0.589)	Moderate Correlation
	AGKQA4 (r=0.652)	Moderate Correlation
	AGKQA5 (r=0.555)	Moderate Correlation
	AGKQA6 (r=0.701)	High Correlation
	AGKQA7 (r=0.547)	Moderate Correlation
	AGKQA8 (r=0.574)	Moderate Correlation
	AGKQA9 (r=0.564)	Moderate Correlation
	AGKQA10 (r=0.640)	Moderate Correlation
	AGKQA11 (r=0.670)	Moderate Correlation
	AGKQA12 (r=0.752)	High Correlation
	AGKQA13 (r=0.815)	High Correlation
	AGKQA14 (r=0.524)	Moderate Correlation
	AGKQA15 (r=0.629)	Moderate Correlation
	AGKQA16 (r=0.619)	Moderate Correlation
	AGKQA17 (r=0.617)	Moderate Correlation
	AGKQA18 (r=0.600)	Moderate Correlation
	AGKQA19 (r=0.679)	Moderate Correlation
	AGKQA20 (r=0.601)	Moderate Correlation
	AGKQA21 (r=0.611)	Moderate Correlation
	AGKQA22 (r=0.792)	High Correlation
	AGKQA23 (r=0.593)	Moderate Correlation
	AGKQA24 (r=0.734)	High Correlation
	AGKQA25 (r=0.587)	Moderate Correlation
	AGKQA26 (r=0.707)	High Correlation
	AGKQA27 (r=0.636)	Moderate Correlation
	AGKQA28 (r=0.524)	Moderate Correlation
	AGKQA29 (r=0.515)	Moderate Correlation
	AGKQA30 (r=0.688)	Moderate Correlation
	AGKQA31 (r=0.589)	Moderate Correlation

The Cronbach's alpha value of the AGKQA questionnaire was greater than 0.690, showing that it was reliable and trustworthy (Table 4).

**Table 4:** Result of the reliability test of the Indonesian version AGKQA questionnaire.

N of Items	Cronbach's Alpha	Result
**31**	0.930	Reliable

### Descriptive Analysis

A descriptive statistical analysis is generally used to illustrate various data characteristics. The questionnaire response items of this study comprised two answers: true (scored 1) and false (scored 0). Table 5 shows the descriptive statistical analysis of the control and intervention groups, namely minimum, maximum, and average values. The minimum, maximum, and average values of the pretest results in the control group were 9, 25, and 19.04±2.56, respectively, and the post-test values were 10, 27, and 18.79±3.59. There was a decrease in the mean values by 0.25, with p=0.47 in the control group. Patient education using conventional media, such as books, posters, flyers, pamphlets, or other printed media, did not significantly improve the levels of knowledge among participants in the control group.

**Table 5:** Results of descriptive statistical analysis of study participants.

Groups		Minimum	Maximum	Mean	P-Value
**Control**	**Pretest**	9	25	19.04±2.56	-0.25 (0.47)
	**Posttest**	10	27	18.79±3.59	
**Treatment**	**Pretest**	13	25	19.11±2.87	4.49 (0.01)
	**Posttest**	16	31	23.60±3.95	

The minimum, maximum, and mean values of pretest scores from the treatment group were: 13, 25, and 19.11±2.87, while post-test scores were 16, 31, and 23.60±3.95. In the treatment group, the mean scores improved by 4.49 (p=0.01). It suggests that the educational content of the AsmaDroid^®^ app significantly improved the levels of asthma knowledge among participants in the treatment group. Table 5 shows the descriptive statistical analysis of pre-test and post-test scores of the AGKQA questionnaire from the control and treatment groups.

### Hypothesis Analysis

A hypothesis test is a statistical method that uses sample information to determine whether the hypothesis is established on the assumption of general parameters [17]. The hypothesis of this study indicates that the asthma self-management educational content of the AsmaDroid^®^ app effectively improved the levels of asthma knowledge among asthmatic patients, which was determined using the independent sample t-test approach.

An independent sample t-test describes a parametric analysis implemented to determine the statistical evidence in two independent groups and their significant differences [18]. In this study, the test was conducted to determine that the differences in the post-test results between the control and intervention groups were significant. Table 6 shows that this study employed homogeneous data that met the requirements for testing with an independent sample t-test. The obtained data were considered significantly different, assuming their p-value was higher than 0.05 [18].

**Table 6:** Results of Independent Sample t-Test.

Groups		Data Variance	Difference in Mean	P-Value
**Control**	**Posttest**	0.071	4.81	0.001
**Treatment**				

The independent sample t-test confirmed that the difference between the post-test scores of the control and treatment groups was 4.81 (p=0.001), meaning that the educational contents of the AsmaDroid^®^ app significantly improved the levels of asthma knowledge.

## DISCUSSION

This study compared the use of apps (AsmaDroid^®^) with conventional learning media, such as books, posters, and other printed media, to improve asthma knowledge among asthmatic patients. To determine the effectiveness of the app, the study utilized an AsmaDroid^®^ app and an Indonesian version of the Asthma General Knowledge Questionnaire for Adults (AGKQA) questionnaire.

This study involved 140 patients from various groups of sex, age, education, mHealth user experience, asthma knowledge levels, and asthma control. Table 2 summarizes the background and demographic information of the participants. The study mostly included females (53.57%; n=75), aged 18–22 years old (67.85%; n=95), undergraduate (78.57%; n=110), and with high levels of asthma control (66.42%; n=93). The levels of asthma control were determined using an Asthma Control Test questionnaire. This questionnaire has 5 question items and response alternatives, namely: well-controlled (with a score of 25), meaning that the symptoms have been under control over the last four weeks, on-target (20–24), meaning that symptoms have been reasonably well controlled, and off-target (<20) meaning that the symptoms were not fully controlled [19]. The characteristics of asthma control levels had a p-value greater than 0.05, meaning that the difference between both groups was not significant.

All study participants (100%; n=140) had experience using mHealth apps. In terms of levels of asthma knowledge, more than half of participants (57.85%; n=81) were categorized as high, meaning that they had a score of AGKQA pre-test greater than or equal to 60. According to a previous study, an AGKQA score greater than or equal to 60 was considered high, while less than 60 was low [15]. Additionally, there were no statistical differences (p>0.05) between groups in terms of sex, age, and level of education (Table 2).

The use of asthma self-management educational content of the app (AsmaDroid^®^) effectively improved the level of asthma knowledge among its users. There was a decrease of 0.25 in the control group between the pretest and the post-test scores, with an average score of 19.04 for the pretest and an average score of 18.79 for the post-test (p=0.474)(Table 1). In the intervention group, the levels of asthma knowledge among the participants significantly improved, with an average score of 19.11 for the pre-test and 23.60 for the post-test (p=0.001). This clearly describes that the use of the asthma self-management educational content of the app significantly improved the level of asthma knowledge of study participants. This finding was in line with a previous RCT study, which reported that educational interventions delivered through smartphones (telemedicine and app) helped improve the quality of life of asthmatic patients. Additionally, it enhances patients' symptom management ability and reduces the burden on patients and their care providers [20]. Another positive RCT study also reported that an information and communication technology-based intervention had a high level of user satisfaction among minority and urban or low-income asthmatics, improving their clinical outcomes [21].

This study also found a significant difference between the average values of both groups, *i.e*., 23.60 for the intervention group and 18.79 for the control group (p=0.001), with a higher score in the intervention group. This finding is in accordance with a previous study utilizing an RCT method, which found improvement in the levels of knowledge among patients receiving education through digital media, including apps [21]. Additionally, another study found that educational interventions using telehealth (*i.e*., mHealth app) were significantly effective in providing health information related to asthma [22].

In 2018, a systematic review assessed the role of mobile phone technology, *i.e*. asthma self-management apps or interactive software systems, in improving the conditions associated with asthma management among asthmatic children and adolescents. The review was comprehensive, and both quantitative and qualitative studies were synthesized. The quantitative studies provided limited evidence that the use of an app might positively affect managing asthma symptoms, asthma self-efficacy, and asthma medication adherence. Furthermore, the qualitative studies concluded that the app could improve and facilitate asthma self-management among children and adolescents [23].

In the future, it is not difficult to imagine that in this digital age, asthma self-management apps can be a convenient and attractive educational platform for asthmatic patients, notably for the younger generation of any population.

The limitations of this study are the unavailability of the asthma self-management app on Google Playstore, making it difficult for the participants to install the app on their smartphones. Additionally, the background of the participants did not vary, which was from undergraduate to doctoral degree holders aged more than 18 years and less than 27. Therefore, the study results might fail to reflect the opinion of asthmatics with lower educational backgrounds and between the ages of 18 and 27. For further research, it is advisable to involve respondents with more varied backgrounds.

## CONCLUSION

A digital intervention using asthma self-management educational content on a smartphone app could significantly improve the levels of asthma knowledge. The descriptive statistical analysis test confirmed that the minimum, maximum, and mean values of pre-test scores from the treatment group were: 13, 25, and 19.11±2.87, while post-test scores were 16, 31, and 23.60±3.95. In the treatment group, mean scores improved by 4.49 (p=0.001). The independent sample t-test confirmed that the difference between the post-test scores of both groups was 4.81 (p=0.001). All the findings of the statistical analysis showed that the educational content of the AsmaDroid^®^ app could significantly improve the levels of asthma knowledge. However, further research is highly required to examine the effectiveness of this educational content in the real clinical world.

## References

[1] Welte T, Groneberg DA (2006). Asthma and COPD. Exp Toxicol Pathol.

[2] Zanella D, Stefanuto PH, Schleich Fl, Louis R, Focant JF (2018). Breath Print for Asthma Phenotyping. https://orbi.uliege.be/handle/2268/230206.

[3] Barnes PJ, Szefler SJ, Reddel HK, Chipps BE (2019). Symptoms and perception of airway obstruction in asthmatic patients: Clinical implications for use of reliever medications. Journal of Allergy and Clinical Immunology.

[4] George L, Brightling CE (2016). Eosinophilic airway inflammation: role in asthma and chronic obstructive pulmonary disease. Ther Adv Chronic Dis.

[5] Nair P, Martin JG, Cockcroft DC, Dolovich M (2017). Airway Hyperresponsiveness in Asthma: Measurement and Clinical Relevance. J Allergy Clin Immunol Pract.

[6] (2007). National Asthma Education and Prevention Program, Third Expert Panel on the Diagnosis and Management of Asthma. Expert Panel Report 3: Guidelines for the Diagnosis and Management of Asthma.

[7] Jonathan D, Campbell P (2017). Implications for Asthma Treatment Strategies in Managed Care. Suppl Featur Publ.

[8] Wilson SR, Farber HJ, Knowles SB, Lavori PW (2011). A randomized trial of parental behavioral counseling and cotinine feedback for lowering environmental tobacco smoke exposure in children with asthma: results of the LET'S Manage Asthma trial. Chest.

[9] Gibson PG, Powell H, Coughlan J, Wilson AJ (2003). Self-management education and regular practitioner review for adults with asthma. Cochrane Database Syst Rev.

[10] Coffman JM, Cabana MD, Halpin HA, Yelin EH (2008). Effects of asthma education on children's use of acute care services: a meta-analysis. Pediatrics.

[11] Guevara JP, Wolf FM, Grum CM, Clark NM (2003). Effects of educational interventions for self management of asthma in children and adolescents: systematic review and meta-analysis. BMJ.

[12] Ramsey RR, Caromody JK, Voorhees SE, Warning A (2019). A Systematic Evaluation of Asthma Management Apps Examining Behavior Change Techniques. J Allergy Clin Immunol Pract.

[13] Ghozali MT, Satibi S, Ikawati Z, Lazuardi L (2021). Asthma self-management app for Indonesian asthmatics: A patient-centered design. Comput Methods Programs Biomed.

[14] Lwanga SK, Lemeshow S, Organization WH (1991). Sample size determination in health studies: a practical manual. https://apps.who.int/iris/handle/10665/40062.

[15] Allen RM, Jones MP (1998). The validity and reliability of an asthma knowledge questionnaire used in the evaluation of a group asthma education self-management program for adults with asthma. J Asthma.

[16] Redman BK (2003). Measurement Tools in Patient Education.

[17] Iliopoulos I, Ananiadou S, Danchin A, Ioannidis JP (2019). Point of View: Hypothesis, analysis and synthesis, it's all Greek to me. Elife.

[18] Gerald B (2018). A brief review of independent, dependent and one sample t-test. International Journal of Applied Mathematics and Theoretical Physics.

[19] Ghozali MT, Dewi PEN Trisnawati (2022). Implementing the technology acceptance model to examine user acceptance of the asthma control test app. Int J Syst Assur Eng Manag.

[20] Culmer N, Smith T, Stager C, Wright A (2020). Telemedical Asthma Education and Health Care Outcomes for School-Age Children: A Systematic Review. Journal of Allergy and Clinical Immunology: In Practice.

[21] Baptist AP, Islam N, Joseph CLM (2016). Technology-Based Interventions for Asthma—Can They Help Decrease Health Disparities?. Journal of Allergy and Clinical Immunology: In Practice.

[22] Brown W, Schmitz T, Scott DM, Friesner D (2017). Is Telehealth Right for Your Practice and Your Patients With Asthma?. Journal of Patient Experience.

[23] Alquran A, Lambert KA, Farouque A, Holland A (2018). Smartphone Applications for Encouraging Asthma Self-Management in Adolescents: A Systematic Review. International journal of environmental research and public health.

